# Navigating the Hidden Curriculum: A Study of Resource-Based and Stories-Based Interventions in Higher Education

**DOI:** 10.3390/bs16020273

**Published:** 2026-02-13

**Authors:** Al Robiullah, Lacey Quadrelli, Leslie Remache, David Reed Akolgo, Gerardo Ramirez, Rebecca Covarrubias, Matthew Jackson, Ji Yun Son

**Affiliations:** 1 Department of Educational Psychology, Teachers College, Ball State University, Muncie, IN 47306, USA; al.robiullah@bsu.edu (A.R.); lbquadrelli@bsu.edu (L.Q.); drakolgo@bsu.edu (D.R.A.); 2 Department of Psychology, Stanford University, Stanford, CA 94305, USA; lremache@stanford.edu; 3 Department of Psychology, University of California, Santa Cruz, Santa Cruz, CA 95064, USA; rebeccac@ucsc.edu; 4 Department of Psychology, California State University, Los Angeles, Los Angeles, CA 90032, USA; mjacks40@calstatela.edu (M.J.); json2@calstatela.edu (J.Y.S.)

**Keywords:** motivation, diversity, wise intervention, achievement

## Abstract

This study examines the effectiveness of difference-education interventions as institutional strategies that support students’ coping during the transition to college. We tested an intervention with two components: a resource-focused approach that makes the hidden rules of higher education explicit, and a student-driven narrative approach featuring unscripted stories from peers describing how they navigated common academic- and life challenges. The study involved 716 first-year students at a Minority-Serving Institution who were randomly assigned by course section to one of the two intervention conditions, with a campus-wide comparison group (N = 2708) drawn from non-participating sections. Results showed significant improvements in Fall-semester GPA and first-year retention for students in both intervention conditions relative to the no-treatment comparison group. Contrary to prior work, first-generation students did not benefit more than their continuing-generation peers. These findings suggest that difference-education interventions may support coping by helping students make sense of academic challenges, anticipate institutional demands, and respond to setbacks with greater persistence. Resource-based and narrative-based approaches may therefore contribute to students’ ability to manage academic difficulty and remain engaged during the early stages of college, particularly in Minority-Serving Institutions.

## 1. Introduction

First-generation college students often face barriers related to the unspoken expectations and institutional knowledge that can be difficult to access without prior exposure to higher education. While past interventions have attempted to surface this hidden curriculum through structured narratives and resource sharing, these approaches often rely on narrowly scripted stories tied to specific identity categories like social class. We expand this approach by incorporating unscripted, student-driven narratives that reflect a wider range of lived experiences, from immigration and parenting to writing anxiety and financial strain. By combining these stories with concrete resource information, and testing their impact on student outcomes through a randomized design in a first-year course, we examine whether this more inclusive and ecologically valid intervention can better support student persistence and success.

### 1.1. The Hidden Curriculum

The hidden curriculum in higher education refers to the unwritten, unspoken, and implicit information and values that govern daily operations (Laiduc & Covarrubias, 2022). It represents the insider knowledge privileged by institutions of higher education (IHEs), perpetuating access and achievement gaps for those not in the know (Ramirez et al., 2021; Giroux & Penna, 1979). The hidden curriculum encompasses academic, social, and cultural expectations about what it means to be a “good student” (Margolis et al., 2001).

These norms include how to initiate and navigate faculty interactions, form study groups, or work through bureaucratic processes that the institution might require for persistence, such as meeting with an academic advisor to clear enrollment holds (Jack, 2015; Collier & Morgan, 2008). Additionally, the hidden curriculum encompasses tacit expectations about time management and professional networking (Smith & Lucena, 2016; Anyon, 1980).

The hidden curriculum presents unique challenges for first-generation college students (hereafter, first-gen students) due to structural inequities in higher education. Institutions often operate on assumptions and hidden rules that align with the experiences of continuing-generation students, inadvertently creating barriers for those without prior exposure to college environments (Pascarella et al., 2004). In other words, the hidden curriculum privileges dominant forms of cultural capital, which include familiarity with institutional norms, comfort interacting with authority figures, and awareness of college expectations (Lareau, 2015) and marginalizes the cultural capital of first-generation students (Goudeau et al., 2024; Covarrubias et al., 2022; Yosso, 2005). As Jehangir (2010) argues, this can lead to confusing and burdensome experiences for first-generation students who have to simultaneously navigate academic learning and decipher implicit institutional norms.

Recognizing this challenge, student-affairs professionals are reimagining their approach to develop more inclusive, proactive, and personalized strategies to make the hidden curriculum explicit (Hockings, 2010). Many universities have implemented first-year experience programs and orientation sessions that explicitly teach campus norms and expectations (Barefoot, 2000). Others have focused on faculty development to increase awareness of the hidden curriculum and promote inclusive teaching practices (Gable, 2021). Additionally, some institutions have created peer mentoring programs to help new students navigate campus culture (Collier, 2017).

### 1.2. Diverse Ways of Doing College

One approach for enhancing the effectiveness of these first-year programs is to design them with the specific needs and backgrounds of the student population being served. When first-generation students’ lived experiences and narratives are incorporated into the classroom climate as legitimate bodies of knowledge, it can amplify their sense of belonging (Jehangir, 2010). But when first-generation students’ cultural wealth is not recognized by institutional structures, it can promote marginalization (Jehangir, 2010).

These studies point to the potential of acknowledging students’ backgrounds to facilitate college transitions. Difference-education interventions are designed to normalize academic struggles by explicitly explaining how students’ social-class backgrounds shape their experiences of college, rather than treating those struggles as individual deficits (Stephens et al., 2014, 2019; Townsend et al., 2021). In Stephens et al. (2014), first-year college students were randomly assigned to hear either narrative-based approach that highlighted students’ or general college advice from experienced peers. Students in the difference-education (i.e., background-linked narrative) condition learned how being the first in their family to attend college led to struggles with imposter syndrome, asking for help, and navigating office hours. This version of the intervention improved academic performance and reduced the achievement gap between first-generation and continuing-generation students. It also increased first-generation students’ use of office hours and comfort with seeking support from faculty. These effects were not observed in the control group, which heard similar stories that did not mention background or social identity. By causally linking social class to navigation challenges, the difference-education intervention helped students reframe their difficulties as understandable, shared, and manageable rather than personal or unique.

Ramirez et al. (2021) examined the impact of a difference-education intervention at a Minority-Serving Institution (MSI) where a majority of students were first-generation. We use MSI to refer to institutions that primarily serve students of color, in contrast to Predominantly White Institutions, PWI. This work extended previous research in two important ways. First, it tested a version of difference-education in a setting where first-generation students were not a small subgroup but instead made up the majority of the student body. Second, the intervention took place in a college environment with fewer institutional resources than those featured in most prior difference-education studies. Thus, the intervention was implemented as a series of videos and interactive questions assigned as homework in a first-year experience course (called Introduction to Higher Education, IHE) taken widely by undergraduate students. Even in this context, where students shared similar social backgrounds, the intervention led first-gen students to obtain higher GPAs and lower dropout rates compared to a no-treatment group These results challenge the assumption that difference-education is most effective when underrepresented students are surrounded by peers from more advantaged backgrounds (e.g., in a PWI). Follow-up studies by Jackson et al. (2025) and Remache et al. (2023) tracked outcomes over five semesters and across multiple cohorts, including those enrolled during the COVID-19 pandemic. The results showed sustained academic benefits for first-gen students.

More recently, Stephens et al. (2024) tested a similar intervention at two MSIs: a public Historically Black College and University (HBCU) and a public Hispanic-Serving Institution (HSI). Their findings add further support for the generalizability of difference-education approaches across different institutional contexts. Like our earlier work, this study found improvements in academic performance among students who received the difference-education intervention. These studies suggest that difference-education is not limited to highly resourced or predominantly white campuses. Instead, it can offer meaningful benefits for students at institutions that already serve large populations of first-generation, low-income, or racially minoritized students.

### 1.3. Current Work

In earlier studies (e.g., Ramirez et al., 2021), we compared a resource-focused video condition with a combined format that included both resource information and scripted difference-education stories. Both conditions led to improved academic outcomes, and the combined format produced the strongest effects. Nevertheles, these interventions still followed the conventional model of difference-education (Stephens et al., 2014), using carefully scripted peer stories that focused specifically on social class and generational status. The student actors featured in the panels did not select or shape the message they delivered, and the interventions emphasized shared challenges linked to a relatively narrow set of identity categories.

We take a more student-driven narrative approach. Instead of relying on pre-written scripts, we invited real students to describe their college experiences in their own words. These unscripted narratives reflect a broader range of social realities. Some students spoke about immigration status, being a single parent while in school, or working multiple jobs. Others described their efforts to support their families or to pursue education despite long-standing financial strain. Still, others shared academic challenges such as writing anxiety, often layered with the difficulty of navigating higher education without guidance from family members. By including these stories, we move beyond the typical difference-education format and place greater emphasis on the complexity of students’ identities and the varied forms of resilience they bring. This approach reflects recent calls to recognize the diversity within the first-generation student population and to avoid flattening their experiences into a single narrative (Ardoin, 2021; Nguyen & Nguyen, 2018).

The intervention extends existing difference-education models by altering how student experiences are represented and integrated. Rather than focusing the intervention narrowly around social class, we centered students’ voices and made space for other identities, intersectional identities, and other background pressures that influence how college is experienced. This approach offers a useful step toward developing interventions that speak to a broader range of students, especially at institutions that serve populations historically underrepresented in academic research.

In addition to including more varied and ecologically valid narratives, we also examined whether combining these stories with resource information would offer added value. Among the sections of the Introduction to Higher Education (IHE) course that agreed to participate, we randomly assigned each section to one of two treatment conditions: a resource-only condition or a combined condition that included both resources and student narratives (what we refer to as Stories videos). Because the intervention was delivered as an online homework assignment, section-level randomization ensured that all students within a given class received the same assignment. These were compared with a no-treatment group, which was composed of students in IHE sections that did not participate in the intervention. We measured academic outcomes including GPA and persistence during students’ first year.

The design of this study allowed us to address three research questions:

- (RQ1) First, do either of the interventions, the resource-only or the student stories plus resources condition, improve academic outcomes compared to a no-treatment group?
- (RQ2) Second, does combining student-driven narratives with resource information provide additional benefits beyond the resource-only condition?
- (RQ3) Third, do the effects of these interventions differ for first-generation and continuing-generation students?

We hypothesized that students in both intervention conditions would show improved academic performance (GPA) and higher retention rates compared to students in the no-treatment condition. We also expected that the stories plus resources condition would produce stronger outcomes than the resource-only condition, particularly for first-generation students. Given the broader range of experiences represented in the narratives, we anticipated that the intervention might benefit continuing-generation students as well, though to a lesser extent. Finally, we expected that the positive effects would be more evident in the Fall semester, given the potential for pandemic-related disruptions to affect Spring outcomes.

## 2. Method

### 2.1. Participants

The intervention was implemented at California State University, Los Angeles (Cal State LA). Our primary study sample consisted of 716 first-year students enrolled in sections of the Introduction to Higher Education (IHE) course that were randomly assigned to one of the two treatment groups: resource-only or combined student stories plus resource intervention. This sample was predominantly Latinx/Hispanic (81.6%), followed by Asian (13.8%), Black (2.5%), White (0.4%), Multiracial (1%), and Unknown (0.7%), reflecting the diverse student body at Cal State LA. The majority of participants were first-generation college students (58%) and women (63.4%). The study was approved by the IRB at Cal State LA [908693-1].

In early planning, our research design included randomly assigning some participating sections to a no-treatment control group to rigorously evaluate the effectiveness of the interventions. We were not able to carry out this plan as campus administrators preferred that all participating sections receive some support through the online homework assignment. To address this constraint, we identified a campus-wide comparison group (N = 2708) made up of students enrolled in IHE sections that did not participate in our study. These students had no exposure to the intervention and their data were obtained in the aggregate. Because of this limitation, there is no individual-level demographic data for this group. Thus, we can only examine the moderating effect of generation status within the sample of 716 students who received one of the two treatment conditions. While this no-treatment comparison group does not allow for perfect matching nor individual-level analysis, it provides a practical baseline for evaluating the effectiveness of these interventions at scale.

### 2.2. Research Design and Procedure

Sections of the IHE course were randomly assigned to either the resource condition or the student-driven Stories + Resources condition. In these two treatment conditions, students completed an online, interactive homework assignment (estimated to take less than 1 h) which was assigned by instructors via the campus Learning Management System. The assignments were Qualtrics links that included a series of videos interspersed with interactive questions. The interventions were implemented during the Fall 2019 semester, and we tracked institutional measures of academic outcomes (e.g., GPA) through the 2019–2020 academic year.

#### 2.2.1. Resource Condition

The resource condition consisted of a set of carefully scripted video lessons designed to address the hidden curriculum by providing detailed information on how and why students should take advantage of key resources offered at the university. The resource homework assignment was composed of four modules that each covered a resource that can contribute to academic success: office hours, study groups, time management, and academic support services (tutoring and writing centers). The office hours module explained their purpose, provided strategies for effectively engaging with professors, and asked students to consider why, when, and how they might use office hours. The study groups module highlighted the benefits of collaborative learning through study groups and offered practical advice on initiating and maintaining these peer-support systems. The time management module introduced the consequences of not managing time well and covered various planning tools (e.g., paper planner, digital calendars) and techniques to help students better organize their academic and personal responsibilities. Lastly, the academic support services module emphasized the value of tutoring and writing centers, which clarified the process of accessing these services, and encouraged students, including those who felt they were struggling as well as those who felt they were succeeding, to utilize these campus resources.

To increase engagement and make the content more concrete, each module incorporated interactive elements and activities. For example, in the time management module, students were asked check for planning apps already installed on their smartphones. In the academic support services module, students were asked to locate different centers on a campus map.

#### 2.2.2. Stories + Resources Condition

Participants in the Stories + Resources group received the same resource modules as described above. Before they engaged in those modules, they had an additional module designed to contextualize these resources within diverse student stories.

Unlike prior difference-education interventions that relied on scripted performances by student actors, this version was developed from interviews with 10 undergraduate students (7 Latina/o, 1 Black, 1 Asian American, and 1 Middle Eastern; 3 male and 7 female) who candidly shared their experiences and perspectives, reflecting the demographic composition of the institution. The narratives represented a broad spectrum of student backgrounds and experiences, intentionally extending beyond social class to include race, ethnicity, immigration status, first-generation status, parenthood (e.g., being a single mother), and intersections of these identities. Students connected these experiences to common challenges faced by many undergraduates, including time management, transportation difficulties, balancing work and school, navigating university bureaucracy, working through cultural differences, and overcoming imposter syndrome. Excerpts from all interviewed students were included in the final video materials and were selected based on their ability to convey a shared message of belonging and persistence in a concise format while preserving the substance of students’ experiences. The narratives were lightly edited for length and clarity but were not scripted or altered to change intended meaning. To ensure accuracy and avoid misrepresentation, students reviewed the final edited clips and expressed approval of the excerpts selected for inclusion. By connecting these personal stories to the challenges of navigating college, the stories module aimed to not only validate participants’ diverse experiences but also contextualize the resource modules. Placing the stories module before the resource modules was intended to help students see how specific resources could be relevant in the context of lived experiences shaped by background and various identities.

### 2.3. Outcome Measures

We obtained institutional data on students’ academic outcomes, GPA and number of credit-baring units completed, for both Fall 2019 and Spring 2020 semesters. These variables served as our main outcomes of interest. We also tracked retention, defined as enrollment in Spring 2020 following enrollment in Fall 2019. Note that the Spring 2020 semester coincided with the onset of the global COVID-19 pandemic, which prompted an emergency shift to remote learning. This disruption likely introduced new stressors and variability into students’ academic experiences and new structural barriers to navigating college. Given this context, our outcome measures capture academic success and persistence across two very different semesters, one right before and right after the onset of the COVID-19 pandemic. The Spring 2020 measures may be interpreted as a measure of how students adapted during a period of great uncertainty and upheaval.

### 2.4. Data Processing

Grades ranged from 0.00 to 4.00 across both terms (Mean = 2.91, SD = 0.88), and the number of units completed ranged from 0 to 20 (Mean = 12.14, SD = 4.01). In some cases, students received a GPA of 0.00 despite being enrolled. These were not treated as missing data, as they indicated that students earned grades too low to receive credit (such as Ds or Fs). We classified students as having dropped out during a term if they had a GPA of 0.00 and no course enrollment in a subsequent term. To examine whether attrition was associated with prior academic performance, we compared the Fall-term GPA for students who later discontinued enrollment to those who remained enrolled. Students who dropped out had significantly lower GPAs in the preceding term (STATS), indicating that attrition was not random with respect to academic performance. At the same time, student departure may occur for multiple reasons, including transfer or other structural factors, and the available data do not allow us to distinguish among these alternative trajectories.

Students who dropped out in one term and did not return in the following term introduced some missing data, affecting about 8 percent of the sample. In this data, students who dropped out likely would have earned lower GPAs if they had remained enrolled. If dropout rates varied across groups, excluding these cases with listwise deletion could artificially inflate GPA estimates for groups with higher attrition. To address this, we used multiple imputation (Newman, 2014) including the following variables as predictors in the imputation model: high school GPA, treatment condition, generational status, Fall GPA, Spring GPA, number of units completed in each term, gender, and race or ethnicity.

## 3. Results

### 3.1. Effect of Interventions on GPA

We were primarily interested in the academic performance of students across the Fall and Spring semesters. Consistent with prior work (Remache et al., 2023), we anticipated that during the upheaval of COVID-19 (i.e., Spring 2020) there would be inflated course grades. Inflated grades could make it difficult to ascertain the benefits of the intervention.

**Figure 1:**
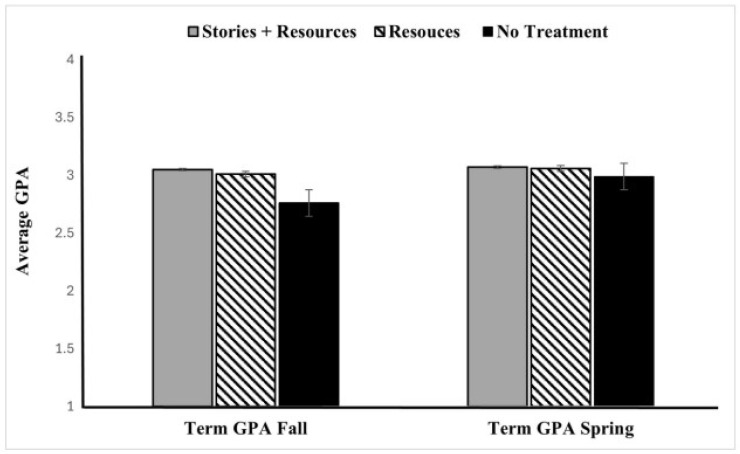
The effect of intervention on GPA across three different group in both Fall and Spring semester.

We begin by presenting results (see Figure 1) that include data for the no-treatment condition which allows for a more robust assessment of the impact of our interventions on student GPA. By comparing the treatment groups to this baseline, we can more accurately determine the effectiveness of the interventions.

To evaluate the impact of the interventions on students’ GPA, we conducted a 3 (Group: Resources Only, Stories + Resources, No-Treatment) × 2 (Term: Fall, Spring) mixed-repeated measures ANOVA. Group was a between-subjects factor and Term was a within-subjects factor, representing GPA across two semesters. There was a significant main effect of term, F(1, 3422) = 18.86, *p* < 0.001, such that GPAs were significantly lower in the Fall than Spring (the COVID-19 term). We also found a significant main effect of group, F(2, 3422) = 13.95, *p* < 0.001. Post hoc Tukey HSD revealed that the mean difference between the Resources group and Stories + Resources group was not significant (*p* = 0.923 > 0.05) but both the Resource (*p* < 0.001) and the Stories + Resources (*p* < 0.001) group were significantly different from the No-Treatment group.

To further explore the significant group × Time interaction observed in the mixed-factor ANOVA, F(2, 3422) = 13.679, *p* < 0.001, we conducted follow-up simple effect analyses (e.g., one-way ANOVAs examining the effect of group on GPA for each term). For the Fall semester, a one-way ANOVA revealed a significant main effect of group, F(2, 3422) = 20.06, *p* < 0.001. A Tukey post hoc test revealed that the Stories + Resources group GPA (M = 3.05, SD = 0.75) and Resources only group (M = 3.01, SD = 0.73), had significantly higher average GPAs than the No-Treatment group (M = 2.76, SD = 0.97). The mean difference between the Resources group and the No-Treatment group was 0.25 (*p* < 0.001), and between the Stories + Resources group and the No-Treatment group was 0.29 (*p* < 0.001). However, the two treatment groups are not significantly different from each other (Mean Difference = 0.0364, *p* = 0.864). For the Spring semester, there was no significant main effect of group (*p* = 0.142).

### 3.2. Effect of Intervention on Retention Rate

**Figure 2:**
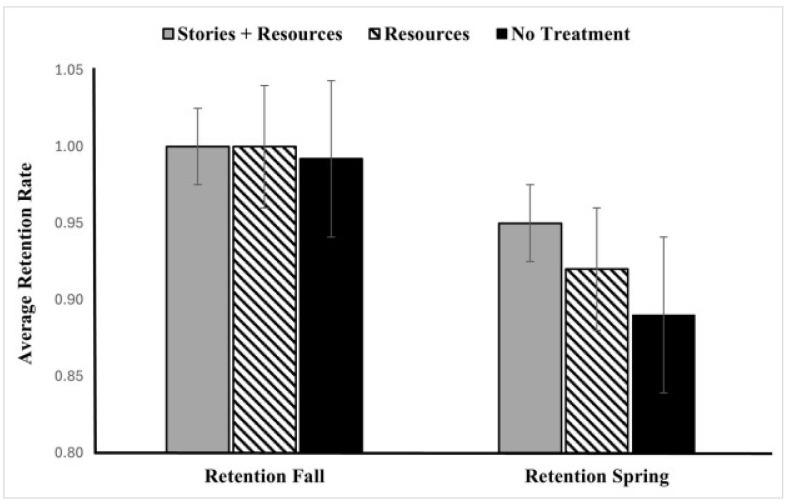
The effect of intervention on retention across three different groups in both Fall and Spring semester.

For retention rate, we once again ran a group × term mixed-factor ANOVA. There was a significant main effect of term, indicating that retention varied between the Fall and Spring semesters, F(1, 3422) = 94.939, *p* < 0.001, with higher retention rates in the Fall compared to the Spring. We also found a significant main effect of treatment, F(2, 3422) = 6.225, *p* = 0.002, indicating that retention rates differed across the three group. The post hoc Tukey test reveals that the mean difference between the Resources group and the Stories + Resources group was not significant (*p* = 0.431). However, the Stories + Resources group was significantly different from the No-Treatment group (*p* = 0.005), while the Resources group did not significantly differ from the No-Treatment group (*p* = 0.120). See Figure 2.

Furthermore, there was a significant interaction between group and time, F(2, 3422) = 4.024, *p* = 0.018. To explore this interaction, we further broke it down by time. For the Fall semester, a one-way ANOVA revealed no significant main effect of group (*p* = 0.137), indicating no substantial difference in retention across group. However, for the Spring semester, a one-way ANOVA revealed a significant main effect of group, F (2, 3422) = 5.493, *p* = 0.004. The Stories + Resources group (M = 0.95, SD = 0.22) had a significantly higher retention rate compared to the No-Treatment group (M = 0.89, SD = 0.31); *p* = 0.008. However, the Resources group (M = 0.92, SD = 0.27) did not significantly differ from either the Stories + Resources group (*p* = 0.423) or the No-Treatment group (*p* = 0.180).

### 3.3. Interaction with College Generation Status

To examine whether intervention effects differed by students’ generational status, we conducted a separate analysis limited to students in the two treatment groups, the subset of participants for whom generation-status data were available. We conducted a 2 (generation status: first-generation vs. continuing-generation) × 2 (group: Resources vs. Stories + Resources) × 2 (term: Fall vs. Spring) mixed-repeated measures ANOVA, with term as the within-subjects factor and generation status and group as between-subjects factors. This analysis examined whether intervention effects on GPA differed by term, group, and generational status. The analysis revealed a significant main effect of Generation Condition, F(1, 712) = 10.72, *p* = 0.001. However, the main effect of time, F(1, 712) = 2.05, *p* = 0.152, and treatment was not significant, F(1, 712) = 0.278, *p* = 0.598. No significant interactions were found between Time and Generation Condition, F(1, 712) = 1.77, *p* = 0.183, or between Time and Treatment group, F(1, 712) = 0.206, *p* = 0.650. Additionally, there was no significant interaction between generation and treatment F(1, 712) = 1.50, *p* = 0.221. Furthermore, the three-way interaction between Time, Generation Condition, and Treatment group was not significant, F(1, 712) = 0.143, *p* = 0.706.

### 3.4. Generation Effect on Retention Rate

We examined the retention rates over two academic terms. The results showed a significant main effect of time, F(1, 712) = 40.65, *p* < 0.001, which indicates that retention rates varied significantly between the Fall and Spring terms. Specifically, retention was higher in the Fall with a mean retention rate of 1.00 (SD = 0.037), compared to the Spring where the mean retention rate was 0.93 (SD = 0.250). There were no significant main effects or interactions related to generational status or other factors (all *p* > 0.05).

## 4. General Discussion

Institutions often operate under the assumption that all students enter higher education with a shared understanding of implicit norms and expectations. Universities implement programs to teach students about the hidden curriculum, but these programs may benefit from acknowledging how background and identity shapes access to and use of institutional resources. Without this acknowledgement, such programs might not be as effective as they can be.

### 4.1. GPA and Dropout Outcomes

The results revealed a main effect of treatment on GPA, primarily observed in the Fall semester. Students in both intervention group (Stories + Resources and Resources only) earned higher GPAs compared to the No-Treatment group. Notably, the two treatment groups were similarly effective in raising academic performance in general.

Another finding worth noting is the variation in treatment effects across time. The impact of the interventions on GPA was stronger in the Fall semester and less pronounced in the Spring. We attribute this reduction to the onset of the COVID-19 pandemic, which significantly disrupted the educational environment. The sudden shift to remote instruction, potential changes in grading policies, and broader uncertainty likely contributed to what has been described as a general GPA inflation during this period (Remache et al., 2023). This widespread rise in grades across all groups may have obscured some of the academic benefits typically associated with the intervention. These findings demonstrate the need to account for external disruptions when evaluating intervention outcomes over time.

In contrast to the GPA results, the analysis of retention revealed a different set of results. The intervention had a significant overall effect on persistence, with this effect emerging more clearly in the Spring semester. Specifically, students in the Stories + Resources group were more likely to remain enrolled than those in the No-Treatment group, while the Resources-only group did not produce a significant difference. The inclusion of student narratives may have offered an added benefit during a period of heightened uncertainty.

Prior work on social-belonging and wise-story interventions shows that peer narratives can help students interpret academic difficulty as common and surmountable by providing points of identification with others who have faced similar challenges (Walton & Cohen, 2011; Yeager et al., 2016). Research on college transition further indicates that peers are a primary source of social and academic information, particularly for first-generation students who may rely less on family-based knowledge about higher education and more on shared sense-making with other students (Cuseo, 1991; Thayer, 2000). In this context, narratives from more experienced peers may function as informal models for navigating institutional barriers, offering concrete examples of how students interpret setbacks, learn unwritten expectations, and persist through challenges (Stephens et al., 2014; Covarrubias et al., 2022). Stories may have supported persistence not only by conveying information about the hidden curriculum, but by shaping students’ relational sense-making, fostering continuity, and reinforcing connections to the institution during a period of disruption.

Although we draw on prior theory to suggest that student narratives may support persistence through processes such as validation, belonging, and meaning-making, these processes were not directly measured in the current study and are offered as theoretically grounded interpretations of the observed outcomes rather than empirically tested pathways.

### 4.2. Outcomes for First- Versus Continuing-Generation Students

Our analysis revealed that both intervention groups, the Resource-only and the Stories plus Resources, were generally effective in improving academic outcomes for all students. This general pattern aligns with prior work demonstrating the value of making the hidden curriculum more accessible through difference-education (Ramirez et al., 2021). However, contrary to prior findings, we did not find that first-generation (FG) students benefited more than their continuing-generation (CG) peers. Instead, both groups showed similar gains, and there were no significant interactions between generation status and intervention groups.

This result stands in contrast to earlier research showing that interventions grounded in students’ lived experiences tend to produce greater benefits for first-generation students (Stephens et al., 2014; Harackiewicz et al., 2016). Typically, these students face more barriers in understanding and navigating academic norms, making them ideal targets for interventions that surface and explain the hidden curriculum. Yet in our study, those differential effects did not appear.

One factor may lie in the structure of the Stories plus Resources group itself. Unlike prior difference-education interventions that emphasized social class or generational status alone, our narratives reflected a wide range of life experiences, including immigration, family responsibilities, work obligations, and aspirations for economic mobility. These stories may have resonated not only with first-gen students but also with continuing-gen students who share similar pressures or who identify with other underrepresented groups. By broadening the scope of identity and experience, our intervention may have addressed challenges that cut across generation status.

Another possible explanation involves the broader institutional context. Our study was conducted at a Minority-Serving Institution that primarily serves commuter students and operates with fewer institutional resources than many four-year residential colleges. At institutions like this, students often spend less time on campus, have limited access to informal peer networks, and may juggle work and family responsibilities alongside their coursework. These structural conditions can reduce opportunities for students to acquire unwritten rules about navigating college, rules that are often transmitted through casual conversations, mentorship, or sustained immersion in campus life. As a result, the kinds of informational and cultural gaps typically associated with first-generation status may be more widespread. When few students have regular access to the social and institutional cues that make the hidden curriculum visible, an intervention that directly names and explains these expectations can offer meaningful support to a broad range of students (see also Murphy et al., 2020). Rather than benefiting only those with a formal first-generation label, the intervention may have addressed more systemic gaps in support, which helps explain why both first- and continuing-generation students showed similar gains.

These findings have broader theoretical implications for difference-education research. Specifically, they suggest that first-generation status may function differently at Minority-Serving Institutions than at the Predominantly White Institutions where much of the prior work has been conducted. Earlier studies often assume that first-generation students are uniquely disadvantaged in access to informal knowledge about navigating college. In MSI contexts, however, navigation challenges may be more widely shared due to commuter structures, limited informal networks, and overlapping work and family responsibilities. Under these conditions, interventions that make expectations explicit may address systemic gaps rather than target a single subgroup, helping to explain why benefits were not concentrated among first-generation students. This pattern identifies institutional context as an important boundary condition for theories of difference-education (see Stephens et al., 2024 as a counter example).

### 4.3. Implications

Our results contribute to the growing body of literature on difference-education and its potential to address educational disparities in higher education. The effectiveness of both intervention groups supports the theory that validating diverse experiences while providing concrete strategies can be particularly powerful in supporting student success (Yosso, 2005; Museus & Quaye, 2009).

Moreover, the sustained effect on retention highlights the potential long-term benefits of early interventions that address the hidden curriculum. These findings suggest that such interventions may help students develop a sense of belonging and self-efficacy that persists even in the face of significant challenges, such as pandemic disruption.

Given the effectiveness of the Stories + Resources intervention, particularly in the crucial first semester, we recommend implementing such treatments as a routine part of the first-year college experience. This proactive approach could help prevent the need for later academic recovery efforts by fostering early engagement with campus resources.

The intervention’s design makes it a cost-effective and scalable option for resource-conscious institutions. It can be readily integrated into existing structures, such as first-year seminar courses, to complement institutional programming. For example, tailored difference-education videos could be incorporated into modules introducing various campus resources, providing both practical information and relatable student experiences.

### 4.4. Limitations and Future Directions

While our study provides valuable insights, it is important to acknowledge several limitations. An important limitation of the present study concerns the nature of the no-treatment comparison group. Although the campus-wide comparison provides a useful benchmark for evaluating the interventions at scale, the absence of individual-level demographic and background data for this group constrains interpretation. Specifically, this limitation precludes matching or adjusting for preexisting differences between treatment- and comparison students and prevents subgroup analyses that could clarify whether effects differ by background characteristics. As a result, comparisons involving the no-treatment group should be interpreted as suggestive rather than definitive evidence of causal impact. In contrast, comparisons between the two treatment conditions benefit from section-level random assignment and therefore allow for stronger causal inference.

Additionally, if we had more time with the students, we would have liked to evaluate more closely how the intervention impacted their approach to and utilization of campus resources. The study, as it currently stands, remains focused on testing whether the intervention influenced academic outcomes rather than examining how students’ understanding of navigation develops over time. A relational, process-oriented approach would treat navigation as an emergent property shaped through ongoing interactions with peers, institutional practices, and cultural resources, independent of immediate outcomes. Investigating such processes would require different methodological approaches, including person-centered analyses, network methods, or qualitative designs that center students’ evolving sense-making. Future work adopting these approaches could provide deeper insight into how students come to understand and enact navigation itself, complementing the causal, variable-centered approach used here.

It is also crucial to note that our interpretations of the differences between continuing-generation (CG) and first-generation (FG) students’ responses to the interventions are ultimately conjectural. While based on the existing literature and our data, these explanations require further empirical validation.

The unexpected impact of the COVID-19 pandemic on our Spring semester data highlights the challenges of longitudinal studies in real-world settings. Future research could benefit from longer-term follow-ups to assess the durability of intervention effects beyond the first year and under more stable conditions.

To address these limitations, future studies could incorporate mixed-methods approaches, including qualitative investigations into how students interpret and apply the stories and resources. This could provide deeper insights into the intervention’s impact and help explain the unexpected findings regarding CG and FG students. Additionally, more frequent assessments of resource utilization and academic behaviors throughout the academic year could clarify the pathways through which these interventions influence student outcomes.

## 5. Conclusions

Our study demonstrates the potential of difference-education interventions in improving academic performance and retention among diverse college students. By making the hidden curriculum more apparent and validating students’ diverse experiences, these interventions can contribute to a more inclusive and supportive educational environment. As institutions continue to grapple with issues of equity and student success, approaches like the one tested in this study offer promising avenues for creating more inclusive and effective learning experiences for all students.

## Data Availability

Restrictions apply to the availability of these data. Data were obtained from Cal State LA and some parts of the data are available by emailing Ji Yun Son with the permission of Cal State LA.
